# Synthesis and Properties of Poly(l-lactide)-*b*-poly (l-phenylalanine) Hybrid Copolymers

**DOI:** 10.3390/ijms150813247

**Published:** 2014-07-29

**Authors:** Marc Planellas, Jordi Puiggalí

**Affiliations:** Departament d’Enginyeria Química, Universitat Politècnica de Catalunya, Av. Diagonal 647, E-08028 Barcelona, Spain; E-Mail: marcplanellas@gmail.com

**Keywords:** biodegradable polymers, block copolymers, self-assembly, polylactide, polyphenylalanine, ring-opening polymerization

## Abstract

Hybrid materials constituted by peptides and synthetic polymers have nowadays a great interest since they can combine the properties and functions of each constitutive block, being also possible to modify the final characteristics by using different topologies. Poly(l-lactide-*b*-l-phenylalanine) copolymers with various block lengths were synthesized by sequential ring-opening polymerization of l-lactide and the *N*-carboxyanhydride of l-phenylalanine. The resulting block copolymers were characterized by NMR spectrometry, IR spectroscopy, gel permeation chromatography, MALDI-TOF and UV-vis, revealing the successful incorporation of the polyphenylalanine (PPhe) peptide into the previously formed poly(l-lactide) (PLLA) polymer chain. X-ray diffraction and DSC data also suggested that the copolymers were phase-separated in domains containing either crystalline PLLA or PPhe phases. A peculiar thermal behavior was also found by thermogravimetric analysis when polyphenylalanine blocks were incorporated into polylactide.

## 1. Introduction

The incorporation of peptide blocks into a synthetic polymer has opened up new challenges in areas as diverse as nanotechnology (e.g., biosensors) and biotechnology (e.g., drug delivery systems, tissue engineering or implants) [1,2,3,4,5]. Peptide blocks can adopt preferred and stable molecular conformations suitable to self-assemble into well-defined molecular arrangements. Specifically, depending on the amino acid side groups, peptides are able to adopt several secondary structures (usually helical or sheet-like morphologies) [6,7]. Feasible phase-separated morphologies depend on the block components, composition, and molecular weight.

Poly(l-lactide) (PLLA) is a well-known polymer used in commodity (e.g., packaging materials and films) and specialized applications such as biomedical devices (e.g., implants and drug delivery systems) because of its degradability in living environments [8]. Furthermore, the monomer can be obtained from renewable resources like starch from either corn or sugar beets. The combination of PLLA with peptide blocks should modify its stability because enzymatic degradation is required to hydrolyze the peptide bonds. Furthermore, the semicrystalline character of PLLA enables the formation of crystallites and amorphous phases depending on the processing conditions, allowing modulation of the PLLA influence on the self-assembly properties of the derived block copolymer.

Although many reports describe the preparation of hybrid copolymers, to the best of our knowledge, very little research activity has focused on block copolymers consisting of polypeptide and biodegradable polyesters [9,10,11,12,13,14]. As representative examples, we can mention the preparation of a hybrid diblock copolymer based on PLLA and poly(γ-benzyl-l-glutamate) (PBLG) [9], the triblock PEG-*b*-PLLA-*b*-PBLG derived from the incorporation of a hydrophilic additional block of polyethylene glycol (PEG) [10] and in general PLLA-*b*-peptide-*b*-PLLA triblocks, where peptides consisting of serine end groups and tryptophane spacer groups are relevant [11]. Block copolymers with peptide blocks having functional side groups also deserve special attention. This is the case of the triblock PLLA-*b*-PGlu-*b*-PLLA copolymer, where the pendant COOH groups of the polyglutamic acid (PGlu) middle block could be easily modified by grafting the RGD peptide (arginine-glycine-aspartic acid) for targeted drug delivery applications [12]. The synthesis of diblock systems like PLLA-*b*-poly(l-lysine) [13] and PLLA-*b*-poly(l-cysteine) [14] has also been reported.

Although many α-aminoacids have found widespread use for hybrid copolymer preparation, little attention has been paid to l-phenylalanine. Thus, while a few works describe the preparation of PEG-*b*-PPhe [15] and PBLG-*b*-PPhe [16] copolymers, the synthesis of a hybrid copolymer with a biodegradable polyester block has never been reported.

The stacking of aromatic ring plays a well-known role to facilitate self-assembling and formation of both chemical and biochemical supramolecular structures [17,18,19]. The restricted geometry and the attractive forces of the aromatic moieties provide order and directionality, as well as the energetic contribution needed for the formation of such well-ordered structures. In particular, Phe-Phe is a widely studied aromatic dipeptide that forms nanotubular structures, as reported by the groups of Gazit [19] and Song [20]. Recently, Tendler *et al.* have reported a new class of Phe-Phe dipeptides with well-defined dendritic structures fabricated on the surface of mica via spin coating [21].

We have recently performed ring-opening polymerization (ROP of lactide using l-phenylalanine (F) or l,l-diphenylalanine (FF) as initiators, which rendered the F-PLLA and FF-PLLA hybrid copolymers, respectively. The PLLA polymer fragment seemed to prefer random coil or helix/strand conformations while the peptide fragment tended to fold. Although the degree of interaction between the two fragments was found to be slightly higher than that reported for other peptide-polymer conjugates, it was small enough to suggest that FF-PLLA is a potential candidate to aggregate forming peptide-guided organization via self-assembly [22].

Herein, we synthesized a new kind of diblock copolymer, poly(l-lactide)-*b*-poly(l-phenylalanine) (PLLA-*b*-PPhe), in order to evaluate its self-assembly properties. Influence of the ratio between the two segment lengths on properties was also evaluated.

The diblock copolymers were prepared using a common strategy based on the preparation of homopolypeptide blocks by ring-opening polymerization (ROP) of protected α-amino acid-*N*-carboxyanhydrides (α-NCAs). To this end, a primary amino end-functionalized polymer was used as initiator [23,24,25]. Specifically, the PLLA-NH_2_ prepolymer obtained by ROP of l-lactide in the presence of stannous octanoate as transesterification catalyst and NH_2_-protected aminopropanol as initiator was considered.

## 2. Results and Discussion

### 2.1. Synthesis of Hybrid Copolymers

**Scheme 1:**
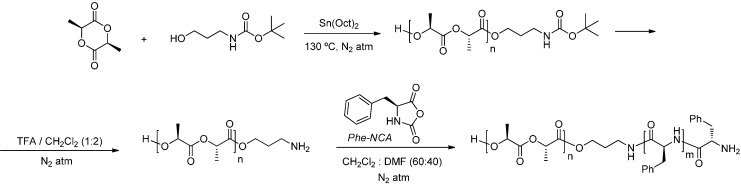
Synthesis for PLLA-*b*-PPhe diblock copolymers.

The synthesis of poly(l-lactide-*b*-phenylalanine) was based on a method first reported by Höcker *et al.* in 1995 [24]. The main point was the preparation of a polylactide end-functionalized with a primary amine group. It is well known that primary amines are good initiators for polymerization of *N*-carboxyanhydrides (NCAs) by ring-opening polymerization (ROP) [23,24,25]. Thus, sequential polymerization of a second block, in our case a peptide sequence of l-phenylalanine, can be easily performed (Scheme 1).

Protected amino end-functionalized poly(l-lactide) (PLLA-NHBoc) was prepared by ring-opening polymerization of l-lactide using *tert*-butyl-*N*-(3-hydroxypropyl) carbamate as initiator and stannous octanoate as catalyst. The reaction was carried out at 130 °C under anhydrous conditions and nitrogen atmosphere for 6 h to avoid any initiation by water molecules, which should lead to undesirable carboxylic acid terminated PLLA. During the reaction, the bulk solution turned into a solid, indicating the polymerization of l-lactide. Once the reaction had been completed, the product was dissolved in CH_2_Cl_2_ and then precipitated by adding cold MeOH. Yields of recovered polymers ranged between 75% and 95%. This could be explained by the formation of low molecular weight oligomers, which were soluble in methanol, and the presence of cyclic oligomers due to backbiting reactions [26,27]. The latter case was confirmed by MALDI-TOF-MS of the methanol soluble fraction. By changing the feed molar ratio of l-lactide to *tert*-butyl-*N*-(3-hydroxypropyl), the degree of polymerization of PLLA-NHBoc (Table 1) could be effectively controlled.

**Table 1:** Molecular weight characterization and degree of functionalization of PLLA-NHBoc homopolylactides.

Sample	*M*_n_^a^	*M*_n_^b^	*M*_W_^b^	PDI ^b^	*M*_n_^c^	*f* ^d^
PLLA_10_	1440	1120	3200	2.62	1285	87
PLLA_25_	3600	3562	6105	1.71	3107	86
PLLA_50_	7200	6978	14,958	2.14	3406	82
PLLA_100_	14,400	13,235	17,217	1.30	8858	73

^a^ Theoretical number average molecular weight determined from the monomer/initiator ratio as: [M]_0_/[I]_0_ × 144; ^b^ Determined from GPC; ^c^ Calculated from the ratio between the areas (*A*) of signals *g* and *e* (see NMR Figure 1) as: (144 × *A_g_*/*A_e_*) + 247. 144 is the repeat unit molecular weight, whereas 247 is the weight associated to the two terminal fragments; ^d^ Functionalization degree percentage calculated from the ratio between the areas (*A*) of signals *h* and *c* or *e* (see NMR Figure 1) as: *f* = 100 × (*A_c_*/2)/(*A_h_*) = 100 × (*A_e_*/2)/(*A_h_*).

**Figure 1:**
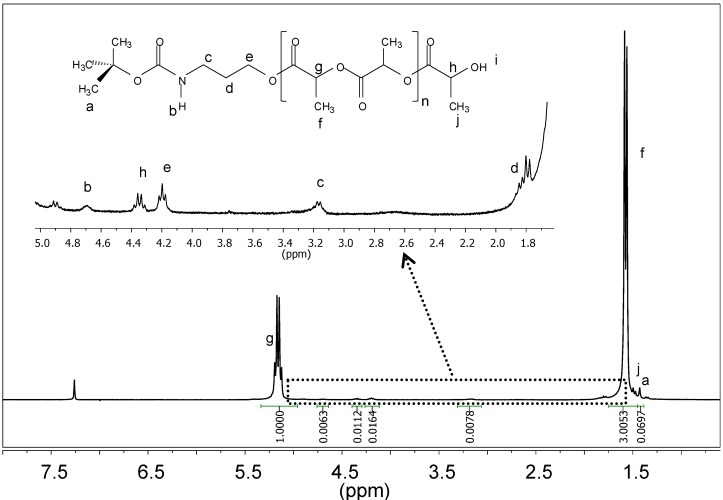
^1^H-NMR spectrum of the PLLA_100_ sample bearing—NHBoc end groups with labeling of observed signals (solvent: CDCl_3_).

The degree of functionalization (*f*) of the polymer was determined by ^1^H-NMR using characteristic signals of the protected amino end group and the lactide terminal group (Figure 1, Table 1). Although functionalization was high in all cases, a significant amount of polymer was not capped with the protected amino terminal group. Some water molecules seemed to remain in the reaction medium—despite the anhydrous conditions used—and were able to act also as initiators of the polymerization, giving rise to carboxylic acid terminated PLLA. Logically, molecular weights were slightly inaccurate when calculated from NMR spectra, assuming complete functionalization. Molecular weights determined from GPC (Table 1) were in full agreement with theoretical values, although the inaccuracies caused by the use of PMMA standards are worth noting.

**Figure 2:**
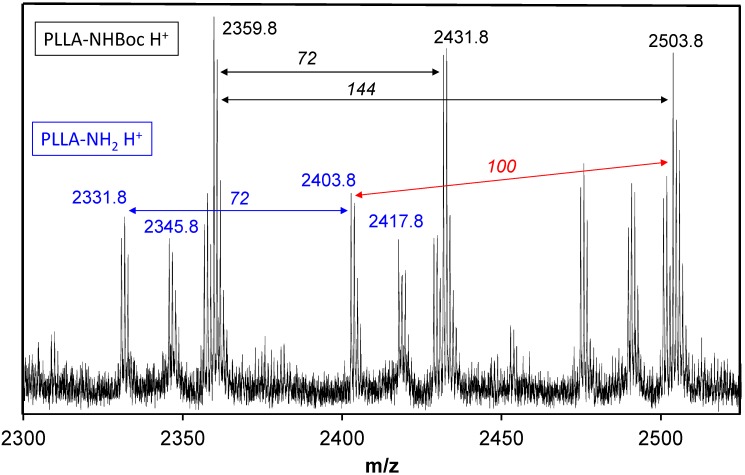
MALDI-TOF-MS spectrum of a mixture of PLLA_25_-NHBoc and PLLA_25_-NH_2_.

Removal of the Boc group of PLLA was carried out by anhydrous trifluoroacetic acid (TFA) treatment (Scheme 1) using a TFA:CH_2_Cl_2_ ratio of 1:2. Although the deprotection proved to be quite efficient, this treatment was left long enough (1 h) to ensure complete removal of Boc groups. Despite this caution, a mixture of PLLA-NHBoc and PLLA-NH_2_ species was obtained, as observed by MALDI-TOF-MS (Figure 2). The value of the mass difference between the two polymers was 100 g/mol, which corresponds to the mass of the protective Boc group. The molecular weights obtained for deprotected samples (PLLA-NH_2_) by MALDI-TOF-MS were always lower than those measured by ^1^H-NMR spectroscopy and GPC (e.g., *M*_n_ ≈ 2800 g/mol was found for the deprotected PLLA_25_ sample, a value that contrasts with those reported in Table 1). This feature is likely due to the overestimation of low-mass poly(l-lactide) chains because of their higher ionization probability [28].

**Figure 3:**
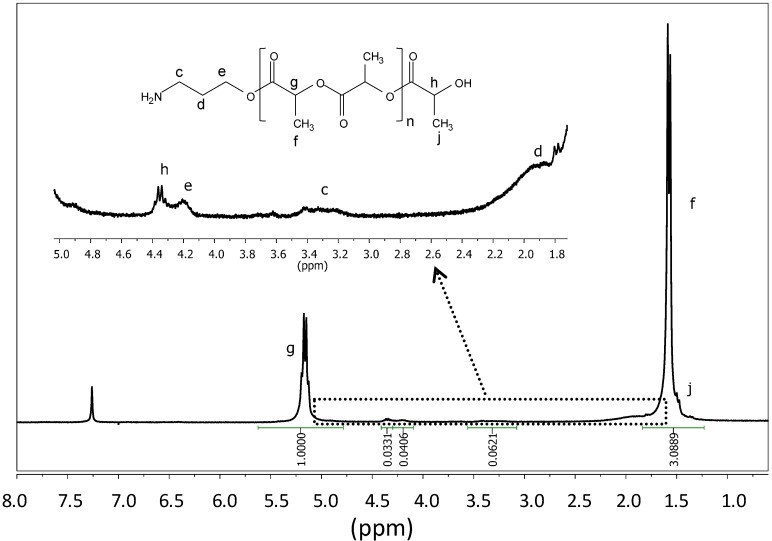
^1^H-NMR spectrum of PLLA_25_ bearing—NH_2_ end groups (solvent: CDCl_3_).

The increase of TFA:CH_2_Cl_2_ ratio to 1:1 was an effective treatment at the indicated conditions since all primary amine functions at the end of the polymer were deprotected with a slight decrease of molecular weight, as confirmed by ^1^H-NMR (Figure 3). Note the complete disappearance of the methyl peak at 1.44 ppm corresponding to the terminal Boc group, whereas the ratio between the areas corresponding to the amine end groups (e.g., peak *e*) and lactide units (e.g., peak *g*) was comparable before and after deprotection.

The amine end group of PLLA was used as a macroinitiator for ring-opening polymerization of l-Phe-NCA (Scheme 1). Gelation of the macroinitiator/monomer solution during the reaction was indicative of the polymerization process of l-Phe-NCA. Several copolymers were prepared varying the composition and chain length of lactide and l-Phe units (Table 2). Deviations of the measured chain lengths from targeted values were due to side reactions (e.g., chain termination or chain transfer), which are inherent to this kind of polymerization. Moreover, it is well known that NCAs are very sensitive monomers [23,24,25]. Thus, this kind of polymerization could proceed via multiple competitive chain growth pathways (different initiating sites and termination reactions), which restrict control over reactivity during polymerization. The ^1^H-NMR results indicate that the length of the peptide block depends linearly on the monomer/initiator ratio. This is a typical feature of primary amine-initiated polymerizations of α-aminoacid *N*-carboxyanhydrides, which are not “living” but facilitate some control of chain length.

**Figure 4:**
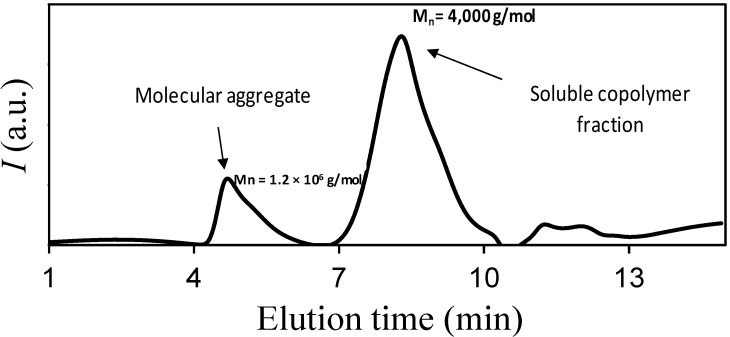
GPC trace of the soluble fraction of the PLLA_25_-*b*-PPhe_13_ sample.

Molecular weights and polydispersity indices of block copolymers could not be determined by GPC due to their low solubility in 1,1,1,6,6,6,-hexafluoroisopropanol (HFIP) and the possible self-assembly of the diblock copolymer [9]. It is interesting to point out that GPC traces of the most soluble samples showed a small peak at very low retention times (Figure 4) that corresponds to *M_n_* values greater than 10^6^ g/mol. This peak could only be explained as a consequence of the aggregation (self-assembly) of the theoretically low molecular weight block copolymers. GPC traces were characterized by a dominant peak indicative of PLLA rich molecules since it appeared at practically the same position as the macroinitiator used in the synthesis. In any case, the ratio between the two indicated peaks is not useful because the chromatogram only corresponds to the soluble fraction, which is less than 20% of the sample, and therefore molecules having larger l-Phe blocks are not considered.

**Table 2:** Molecular weight characterization of block PLLA-*b*-PPhe copolymers.

Sample ^a^	LLA:l-Phe ^b^	LLA:l-Phe ^c^	*M_n_* of the PPhe Block	
			*M*_n_^d^	*M*_n_^c^
PLLA_50_-*b*-PPhe_10_	83:17	83:17	1470	1506
PLLA_50_-*b*-PPhe_40_	55:45	65:35	5880	3675
PLLA_25_-*b*-PPhe_5_	83:17	89:11	735	458
PLLA_25_-*b*-PPhe_13_	65:35	79:21	1911	958
PLLA_10_-*b*-PPhe_25_	28:72	24:76	3675	4617
PLLA_10_-*b*-PPhe_40_	20:80	15:85	5880	8207

^a^ Polymerizations were performed in closed vessels (10 mL) using 0.04 mmol of PLLA-NH_2_ in 2.5 mL of an anhydrous mixture of CH_2_Cl_2_/DMF (60:40) when PLLA block was large (*i.e.*, PLLA_50_ and PLLA_25_), whereas 0.06 mmol of PLLA-NH_2_ in 2.5 mL of an anhydrous mixture of CH_2_CL_2_/DMF (40:60) was used for preparing PLLA_10_-*b*-PPhe_25_ and PLLA_10_-*b*-PPhe_40_ samples; ^b^ Molar ratio between lactide and l-Phe units in the feed reaction medium; ^c^ Calculated from the ratio between signals *a* and *c* (Figure 6a) and Equation (1) that is later explained; ^d^ Theoretical value determined from the used monomer/macroinitiator ratio.

**Figure 5:**
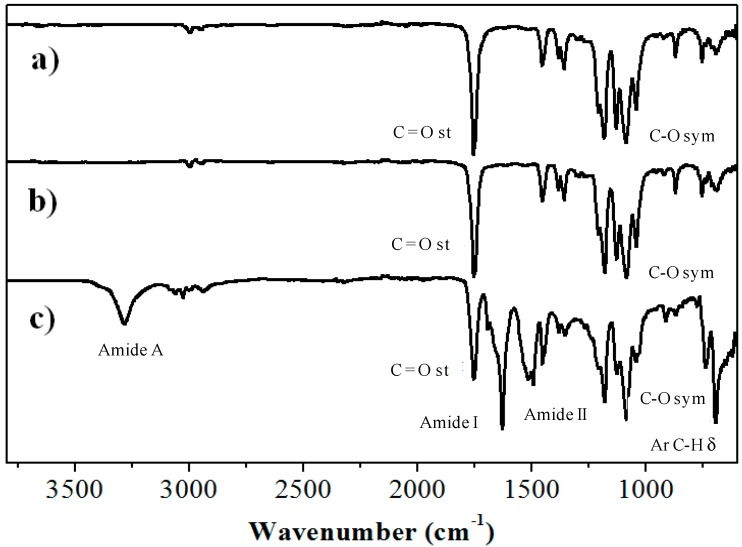
IR spectra of PLLA_25_-NHBoc (**a**); PLLA_25_-NH_2_ (**b**); and PLLA_25_-*b*-PPhe_13_ (**c**).

The IR spectra of PLLA-NHBoc, PLLA-NH_2_ and PLLA-*b*-PPhe are illustrated in Figure 5. Characteristic bands of PLLA (e.g., 1753 cm^−1^ (C=Ο st) and 1183 cm^−1^ (C-O st)) could be detected in all cases, whereas specific signals indicative of the formation of peptide blocks were observed in the copolymer sample. Thus, amide A, amide I and amide II bands were found at 3284, 1631 and 1522 cm^−1^, respectively, together with characteristic signals of phenyl groups at 740 and 696 cm^−1^.

Samples were practically insoluble in CHCl_3_, which is a good solvent for PLLA. This feature is highly important since it demonstrates a change in solubility characteristics and points to an effective block copolymerization reaction. Addition of TFA allowed disruption of hydrogen bonding interactions between amide groups of the peptide sequence, making possible solubilization. It is also interesting to point out that the PLLA block slightly enhanced solubilization of the peptide block because PPhe is practically insoluble in all common solvents for higher numbers of amino acid units.

**Figure 6:**
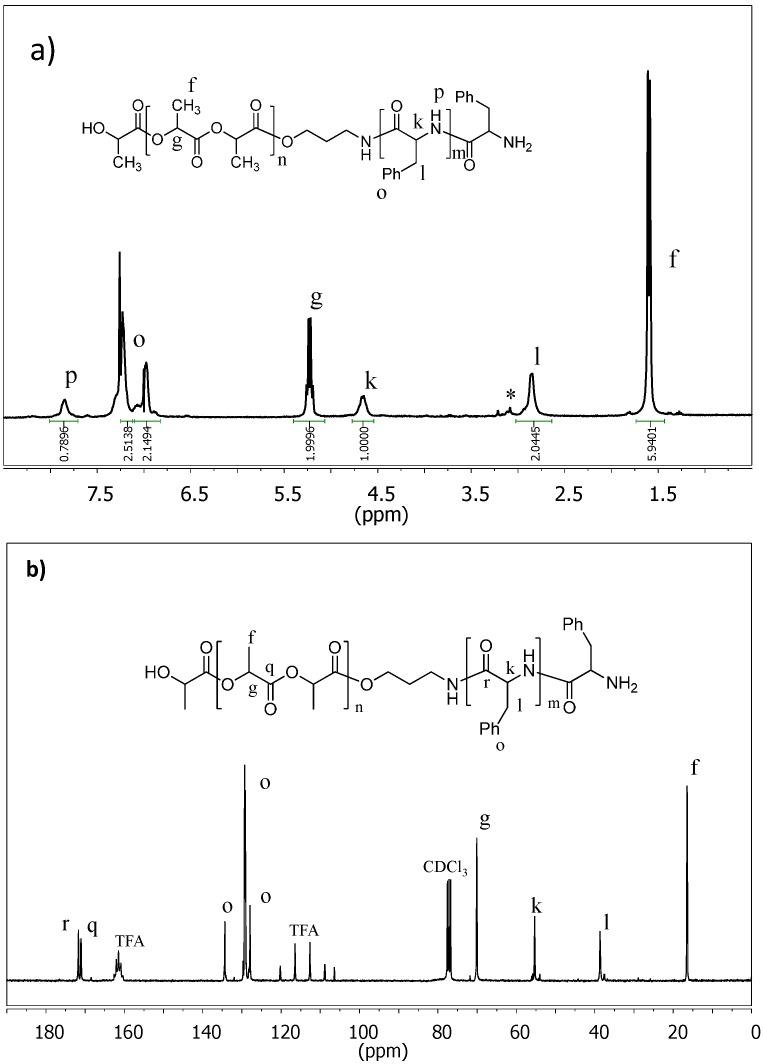
.^1^H-NMR (**a**) and ^13^C-NMR (**b**) spectra of PLLA_50_-*b*-PPhe_40_ (solvent: CDCl_3_/TFA).

The ^1^H- and ^13^C-NMR spectra of all samples were in full agreement with the chemical structure of the synthesized polymers, with no unexpected signals being detected. Spectra clearly indicated the successful incorporation of the peptide block, as shown in Figure 6 for a representative sample. Besides the signals of PLLA, new peaks associated with the l-Phe residues appeared in the ^1^H-NMR (Figure 6a). Specifically, those observed at 7.2 and 6.9 ppm were attributed to the aromatic protons, whereas those at 7.8, 4.6, and 2.9 ppm were assigned to NH, CH and CH_2_ protons, respectively. The two types of carbonylic carbons are also well differentiated in the ^13^C-NMR spectrum (Figure 6b), where peaks at 171.7 (l-Phe unit) and 170.9 ppm (LLA unit) are detected.

^1^H-NMR spectra allowed determining the composition of copolymers from the areas of CH protons of LLA and l-Phe units that appeared at 5.23 and 4.75–4.58 ppm, respectively. Experimental compositions summarized in Table 2 were in reasonable agreement with the monomer feed ratio, although the fit seemed to worsen for a given PLLA-NH_2_ macroinitiator when the PPhe content increased.

From the integral ratio of peaks representative of LLA and l-Phe units (*i.e.*, CH protons at 5.23 and 4.75–4.58 ppm), the number average molecular weight of the polypeptide block (*M_n_*(PPhe)) was evaluated according to Equation (1), which also takes into account the previously deduced degree of polymerization of the PLLA block (*DP*_PLLA_) and the weight of the l-Phe unit (*i.e.*, 147 g/mol):

*M_n_*(PPhe) = 147 × *DP*_PLLA_ × 2 × *A*_4.75-4.58_/*A*_5.23_ (1)

Deduced molecular weights deviated slightly from those expected from the monomer/macroinitiator ratio, as summarized in Table 2, and logically reflect the deviation of copolymer composition with respect to the feed ratio. The molecular weight of the peptide block was generally lower than expected for samples from high molecular weight macroinitiators (e.g., PLLA_50_ and PLLA_25_), which suggests their lower capability to initiate polymerization of l-Phe-NCA rings.

**Figure 7:**
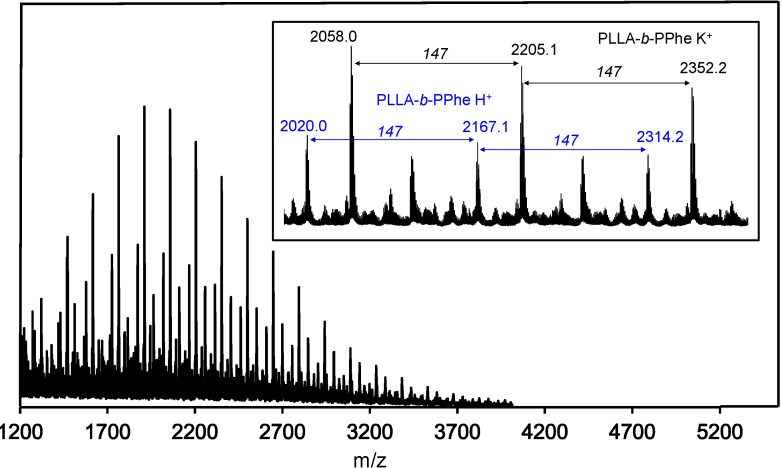
MALDI-TOF-MS spectra of PLLA_25_-*b*-PPhe_13_. The inset shows a partial enlargement.

Block copolymers were also characterized by MALDI-TOF-MS (Figure 7). The obtained mass spectra revealed well-resolved signals with a mass difference between signals of 147 g/mol. This value exactly corresponds to the mass of l-Phe residues, and consequently demonstrates again the polymerization of l-Phe-NCA rings. Two mass peaks observed in the spectra indicated the masses of copolymers charged with a proton or a potassium cation.

### 2.2. Physical Properties of Hybrid Copolymers

Powder X-ray diffractograms of the synthesized hybrid copolymers showed characteristic peaks associated with the crystallization of each constituting block, as depicted in Figure 9 for some representative samples. Thus, typical Bragg reflections associated with the α-form of polylactide (10_7_ helical conformation) could always be observed. Unit cell parameters of this form have been reported by different authors [29,30,31,32] and correspond to an orthorhombic lattice with dimensions in the intervals *a* = 1.078–1.075, *b* = 0.645–0.604 and *c* (chain axis) = 2.78–2.88 nm. Thus, the most intense reflections observed at 0.547, 0.470, and 0.403 nm could be well indexed as the (200) + (110), (203) and (015) reflections of this polymorphic form. PLLA can also crystallize in a second structure constituted by 3_1_ helices named β-form and defined by a trigonal unit cell with *a* = *b* = 1.052 nm and *c* = 0.88 nm [33,34]. This form is usually produced under stress [30] and can be clearly discarded from the experimental X-ray profiles of the studied powder samples since its strong (110) reflection (*i.e.*, that corresponding to 0.526 nm) was not envisaged.

**Figure 8:**
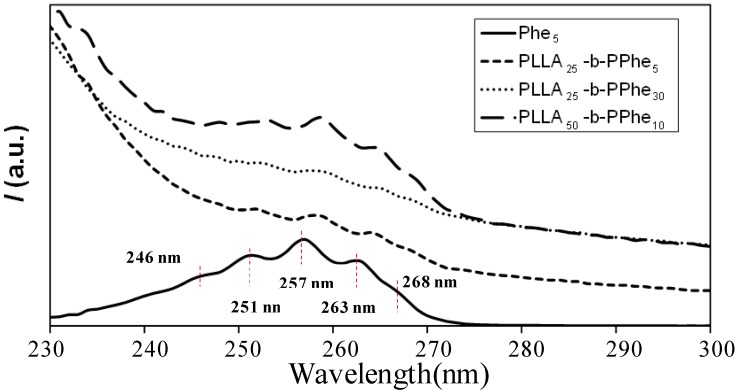
UV-vis spectra of l-Phe and representative hybrid copolymers with different PLLA and PPhe block lengths obtained from 1,1,1,3,3,3,-hexafluoroisopropanol solutions.

**Figure 9:**
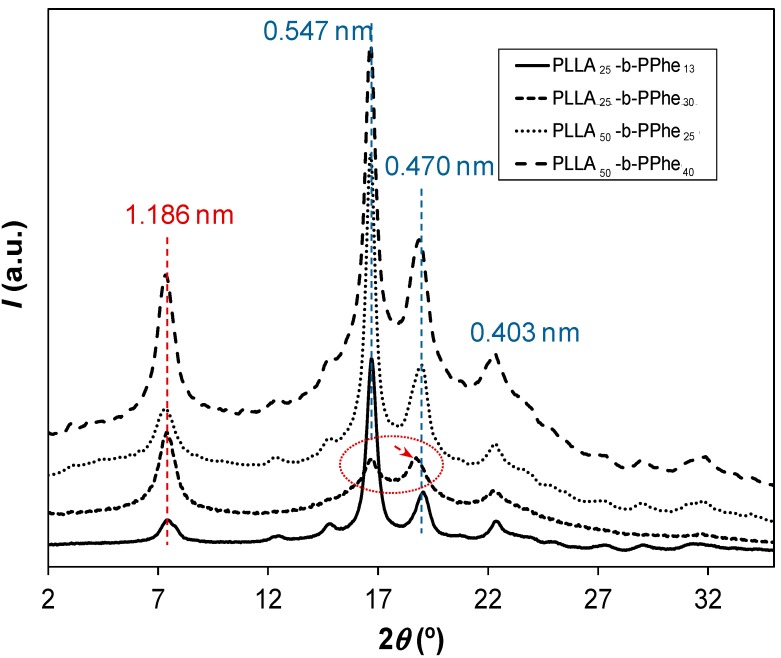
Powder X-Ray diffraction profiles of representative hybrid copolymers having blocks with different lengths.

Patterns of hybrid copolymers show an additional reflection at 1.186 nm with variable relative intensity compared to the characteristic PLLA peak at 0.547 nm that increased with the ratio between the lengths associated with PPhe and PLLA blocks. The indicated spacing is larger than expected for the chain axis period of a polypeptide in the typical *2_1_* helical conformation of a β-sheet form (*i.e*., 0.70 nm) [35], and is probably linked to the intersheet spacing that should accommodate the voluminous aromatic groups. This signal is relevant for the PLLA_25_-*b*-PPhe_30_ copolymer, which in addition shows a shift of the PLLA peak corresponding to the (203) reflection towards a value close to 0.477 nm. It is significant that the intensity of this peak became close to and even higher than that corresponding to the most intense reflection of PLLA (see ellipse in Figure 8), suggesting the additional contribution of a reflection corresponding to the PPhe structure (e.g., that associated with the typical spacing between hydrogen bonded chains, see arrow in Figure 9).

X-ray diffraction profiles also revealed the presence of amorphous halos with an intensity that changes with block length and composition. Thus, the amorphous contribution is clearly higher for the PLLA_50_-*b*-PPhe_40_ copolymer than for the PLLA_25_-*b*-PPhe_13_ sample. Note in the second case that the PLLA block can easily crystallize due to its relatively low size and that its PPhe content is low, and consequently may have a minor disturbing effect during the crystallization process.

Effective polymerization of l-Phe-NCA by using PLLA-NH_2_ macroinitiators was also confirmed by UV-Vis spectroscopy. The spectra were characterized by the presence of absorption maxima at wavelengths close to 246, 251, 257, 263 and 268 nm (Figure 9), which could be attributed to the π-system of the aromatics groups of l-Phe units. The intensity of these peaks decreased with the length of the peptide block, probably because of increasing insolubility. Therefore, only a small fraction of polymer was solubilized in the solvent used (*i.e.*, 1,1,1,3,3,3-hexafluoroisopropanol) when the hybrid copolymer had long peptide blocks. Nevertheless, it is meaningful that the indicated peaks were clearly detected in the more soluble PLLA_25_-*b*-PPhe_5_ sample and appeared at practically the same position as the l-Phe monomer used as a reference. The influence of the PLLA block on the conformation of the peptide fragment of the conjugate seems relatively small since PLLA tends to organize independently, which is a prerequirement for the formation of peptide-guided assemblies. However, the high crystallinity of PLLA can interfere with the peptide assembly because it is well known that simultaneous polymer crystallization and peptide organization become less likely [4].

**Figure 10:**
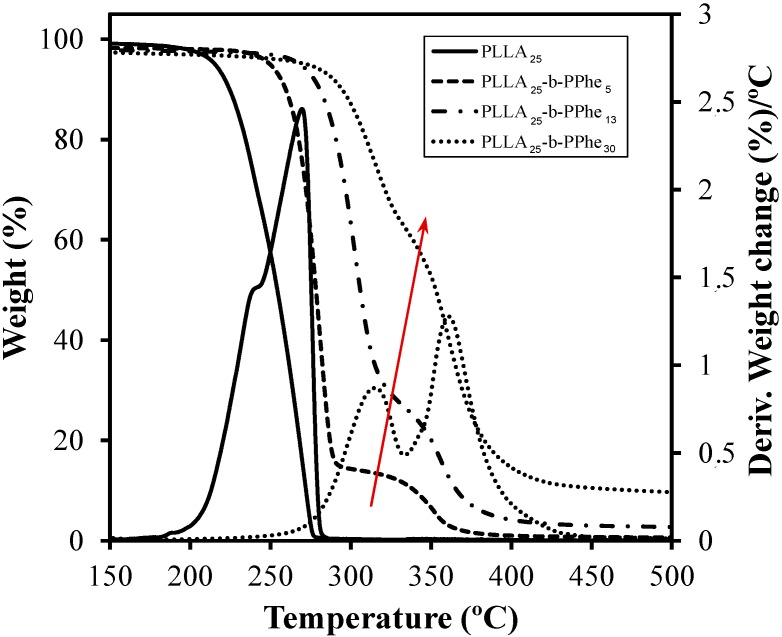
Thermogravimetric and representative derivative thermogravimetric curves of PLLA_25_-NH_2_ and copolymers having different PPhe block lengths.

Thermal degradation behavior of hybrid copolymers is singular, as shown in the TGA traces depicted in Figure 10. In general, two degradation processes can be clearly observed and well correlated with composition. Thus, the first process corresponds to the degradation of PLLA blocks and becomes less important as the PPhe content increases.

The small PLLA homopolymer degraded at the lowest temperature, which is indeed lower than reported for conventional PLLA samples, probably because of the abundant NH_2_ terminal groups. It is well known that degradation of PLLA follows a complex process with the participation of at least two different mechanisms. The main degradation process has been associated with non-radical backbiting ester interchange reactions [27,36,37], whereas the less predominant process that occurred at the beginning of degradation mainly involved the decomposition of terminal groups.

It is interesting to point out that incorporation of PPhe blocks had a significant effect since they hindered the thermal degradation of small PLLA blocks. Note that the initial decomposition temperature and the first peak of the derivative thermogravimetric curve gradually shifted to higher values (*i.e*., from 208.6–281.2 °C and from 269.3–315.1 °C, respectively) as the PPhe content increased. It is also remarkable that degradation of PPhe blocks was enhanced for higher PLA contents (see red arrow in Figure 10) as the greater number of PLLA degradation products could have accelerated the degradation of the peptide block.

**Figure 11:**
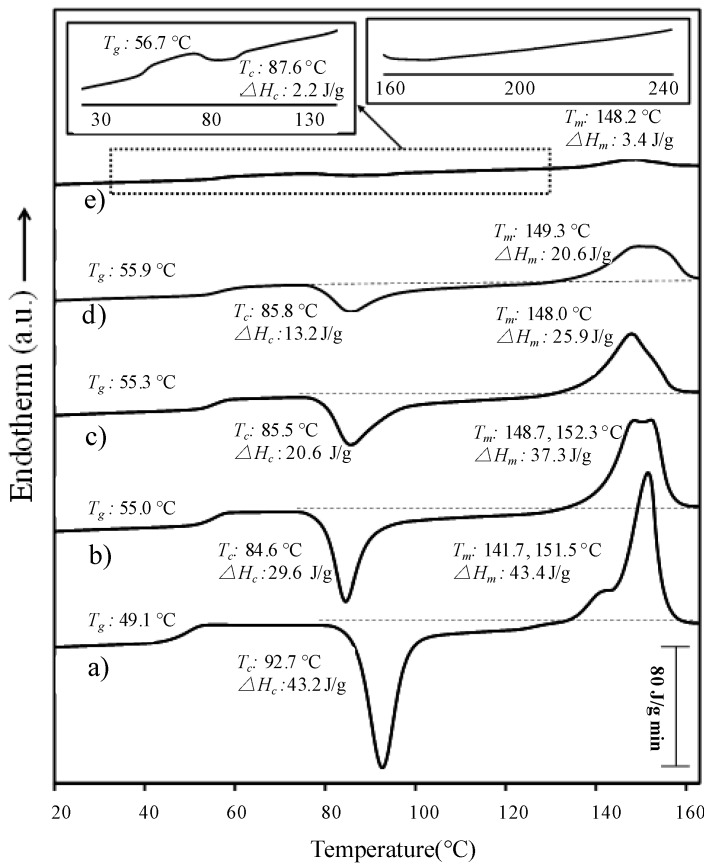
Calorimetric curves of the third heating run (20 °C/min) of PLLA_25_-NH_2_ (**a**) and block copolymers with different chain length units, PLLA_25_-*b*-PPhe_5_ (**b**); PLLA_25_-*b*-PPhe_13_ (**c**); PLLA_50_-*b*-PPhe_25_ (**d**) and PLLA_25_-*b*-PPhe_30_ (**e**). Insets show a magnification of the low temperature region for PLLA_25_-*b*-PPhe_30_ and the DSC trace recorded in the 160–245 °C range for the PLLA_50_-*b*-PPhe_25_ sample.

Thermal properties of new hybrid copolymers are summarized in Table 3, whereas representative DSC traces are shown in Figure 11. Several points merit discussion:

(a) Although peptide blocks gave rise to crystalline arrangements, only melting transitions associated with PLLA blocks were observed, as proved from X-ray diffraction data. Therefore, thermal decomposition seemed to occur before the melting temperature of PPhe was reached.
(b) Melting of PLLA blocks was defined by two well differentiated peaks associated with a lamellar reorganization. The high temperature peak corresponds to recrystallized/reordered lamellae formed during the heating rate. The evolution observed for the copolymers indicated that this reorganization became less significant as the length of the PPhe block increased.
(c) Glass transition temperature slightly increased with the PPhe content (*i.e.*, from 49.1–56.7 °C for the PLLA_25_ series), indicating that some rigid l-Phe units were incorporated in the PLLA amorphous phase. No transition was detected for PPhe blocks, suggesting that they formed crystalline aggregates. Although an increase in the glass transition temperature may also be justified by the molecular weight increase caused by the incorporation of peptide blocks, the effect does not seem highly relevant when *T_g_*s of PLLA_50_-*b*-PPhe_25_ and PLLA_25_-*b*-PPhe_13_ samples are compared (*i.e.*, 55.9 *versus* 55.3 °C).
(d) PLLA melting enthalpy logically decreased when the PPhe content in the sample increased. This reduction is higher than expected according to the copolymer composition (e.g., the melting enthalpy changed from 43.4–3.4 J/g when a block of 30 l-Phe units was incorporated in the PLLA_25_ block, while this enthalpy should be close to 20 J/g according to the real weight of PLLA in the copolymer). Therefore, the presence of peptide blocks hindered crystal growth of PLLA and the sample became more amorphous, as previously deduced from X-ray diffraction data.
(e) PPhe blocks may act as effective nucleation agents since the cold crystallization peak observed for hybrid copolymers was shifted towards lower temperatures compared to that of the PLLA homopolymer. It is also significant that the difference between melting enthalpy and crystallization enthalpy of copolymers was higher than that found for PLLA homopolymers. This suggests an enhanced crystallization during the previous fast cooling run performed from temperatures higher than the PLLA melting temperature. Note that the crystalline PPhe domains were not melted to prevent degradation, and were therefore ideal nucleating agents.
(f) Crystallinity slightly decreased with the molecular weight of the sample for a given composition (e.g., clear differences are found between DSC traces of PLLA_50_-*b*-PPhe_25_ and PLLA_25_-*b*-PPhe_13_ samples in Figure 11).

**Table 3:** Calorimetric data (20 °C/min) of hybrid copolymers obtained in the heating run of a previously quenched sample.

Sample	*T_g_* (°C)	*T_c_* (°C)	*∆H_c _* (J/g)	*T_m_* (°C)^a^	*∆H_m_* (J/g)
PLLA_25_-NH_2_	49.1	92.7	43.2	141.7, 151.5	43.4
PLLA_25_-*b*-PPhe_5_	55.0	84.6	29.6	148.7, 152.3	37.3
PLLA_25_-*b*-PPhe_13_	55.3	85.5	20.6	148.0	25.9
PLLA_25_-*b*-PPhe_30_	56.7	87.6	2.2	148.2	3.4
PLLA_50_-*b*-PPhe_10_	59.6	86.3	21.2	153.9	26.4
PLLA_50_-*b*-PPhe_25_	55.9	85.8	13.2	149.3, 152.4	20.6
PLLA_50_-*b*-PPhe_40_	54.1	86.3	4.1	152.8	5.7

^a^ When multiple peaks are observed, the most intense ones are indicated by bold numbers.

**Figure 12:**
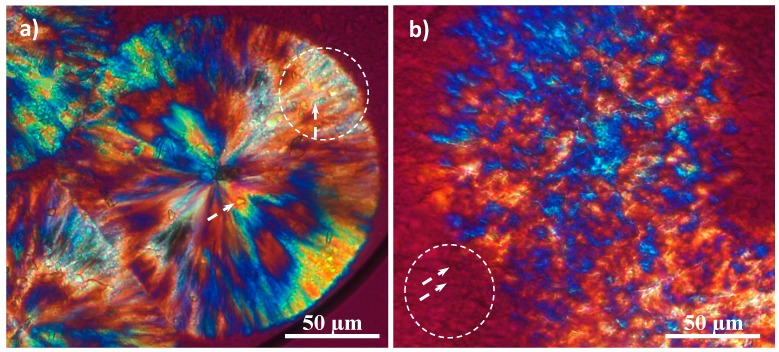
Polarizing optical micrographs showing spherulitic morphologies of the representative PLLA_25_-*b*-PPhe_13_ sample obtained during isothermal crystallization at 132 °C (**a**) and 135 °C (**b**). Circles point out domains where aggregates (white arrows) could be well observed.

Polarizing optical micrographs (Figure 12) also demonstrated the capability of hybrid copolymers to crystallize from temperatures above the PLLA melting temperature. Spherulitic morphologies were always observed, with texture and optical properties being dependent on the crystallization temperature. Thus, at low degrees of supercooling, spherulites did not have a well-defined birefringence and great domains, probably constituted by flat-on lamellae, were observed (Figure 12b). In addition, phase separation and aggregates could be envisaged in areas where crystallization was not yet completed (see white circle and arrows). Spherulites had a typical fibrillar texture and negative birefringence when crystallization was performed at lower temperatures (Figure 12a). Nevertheless, birefringence was still unclear because an incipient banding as well as aggregates associated with peptide rich fractions were detected (see white circle and arrows). These aggregates could act as nucleating agents and should be occluded inside the spherulites due to covalent bonds between PPhe and PLLA blocks.

## 3. Experimental

### 3.1. Materials

l-lactide (LLA) was purchased from Aldrich and recrystallized three times from ethyl acetate before being stored under nitrogen atmosphere. Anhydrous trifluoroacetic acid (TFA) was purchased from Scharlau and used as received. Sn(Oct), *tert*-butyl-*N*-(3-hydroxypropyl) carbamate and triphosgene were provided by Sigma and used as received. Triphosgene was stored at 5 °C. l-phenylalanine was purchased from Bachem and used as received. l-Phe-NCA was prepared from the corresponding α-amino acid and triphosgene in THF at 40 °C under nitrogen atmosphere by a previously described method [10,11].

### 3.2. Polymerization of l-lactide

All experiments were carried out under nitrogen atmosphere in a flame-dried round-bottom flask charged with a magnetic stir bar. PLLA was obtained by ring-opening polymerization of l-lactide initiated by *tert*-butyl-*N*-(3-hydroxypropyl) carbamate (3-(Boc-amino)-1-propanol) and catalyzed by Sn(Oct)_2_. Reactions were performed using a relative low lactide/initiator ratio that was varied from 10–100 in order to obtain PLLA samples with theoretically low molecular weights between 1440 and 14,400 g/mol. Polymers are named by indicating the used monomer/initiator ratio as subscript (e.g., PLLA_10_, PLLA_25_, PLLA_50_, PLLA_100_), which should correspond to the number of lactide units incorporated into the homopolymer chain (*i.e.*, degree of polymerization, DG, of PLLA).

Synthesis of PLLA_100_ is given as an example of the procedure. Specifically, 10 g of l-lactide (69.4 mmol), 475 µL of *tert*-butyl-*N*-(3-hydroxypropyl) carbamate (2.8 mmol) and 40 µL of Sn(Oct)_2_ (0.01 mmol) were introduced to the flask. After being vacuumed and purged with nitrogen three times, the flask was sealed and immersed in a silicone bath at 130 °C for 6 h. The obtained white solid was purified by precipitation from a CH_2_Cl_2_ solution by addition of MeOH.

Yield: 9.56 g (96%). ^1^H-NMR (300 MHz, CDCl_3_): δ = 5.15 (q, *J* = 7.0 Hz, 89H, -C*H*CH_3_), 4.73 (s, 1H, -N*H*), 4.34 (q, *J* = 7.0 Hz, 1H, -C*H*CH_3_), 4.24–4.16 (m, 2H, O-C*H*_2_CH_2_CH_2_-N), 3.37–3.10 (m, 2H, O-CH_2_CH_2_C*H*_2_-N), 1.87–1.81 (m, 2H, O-CH_2_C*H*_2_CH_2_-N), 1.57 (d, *J* = 7.1 Hz, 267H, -CHC*H*_3_), 1.48 (d, *J* = 7.1 Hz, 3H, -CHC*H*_3_), 1.42 (s, 9H, -C(C*H*_3_)_3_). IR (ATR) (ν, cm^−1^): 2998 (CH_3_ st), 2949 (CH_3_ st), 1756 (C=O st), 1455 (CH_3_ δ), 1360 (CH_3_ δ), 1183 (C-O st), 1089 (C-C vibration), 1044 (C-C vibration).

### 3.3. N-Boc Deprotection of Polylactide

The methodology for the deprotection of PLLA_25_ is described as an example of the procedure. Polylactide (2.5 g) was placed in a dried flask and the system vacuumed and purged with nitrogen three times; methylene chloride (30 mL) was used as solvent. After complete dissolution, a large excess of anhydrous trifluoroacetic acid (30 mL) was added. The solution was stirred at room temperature for 1 h, after which time all solvents were rotavaporated. The polymer was redissolved in CH_2_Cl_2_ and the solution was washed with aqueous NaHCO_3_ (5%) and water. After drying over anhydrous Na_2_SO_4_, the solvent was evaporated under vacuum to give a white solid. The product was purified by precipitation from a CH_2_Cl_2_ solution by addition of MeOH.

Yield: 2.03 g (83%). ^1^H-NMR (300 MHz, CDCl_3_): δ = 5.18 (q, *J* = 7.1 Hz, 24H, -C*H*CH_3_), 4.36 (q, *J* = 6.9 Hz, 1H, -C*H*CH_3_), 4.26–4.13 (m, 2H, O-C*H*_2_CH_2_CH_2_-N), 3.45–3.18 (m, 2H, O-CH_2_CH_2_C*H*_2_-N), 2.01–1.82 (m, 2H, O-CH_2_C*H*_2_CH_2_-N), 1.59 (d, *J* = 7.1 Hz, 72H, -CHC*H*_3_), 1.49 (d, *J* = 7.1 Hz, 3H, -CHC*H*_3_). IR (ATR) (ν, cm^−1^): 2998 (CH_3_ st), 2947 (CH_3_ st), 1756 (C=O st), 1455 (CH_3_δ), 1360 (CH _3_δ), 1183 (C-O st), 1089 (C-C vibration), 1043 (C-C vibration).

### 3.4. Polimerization of l-Phe-NCA

Copolymers having different PLLA and PPhe block lengths were synthesized using different ratios between deprotected PLLA and the l-Phe-NCA monomer. In the abbreviated form, copolymers are named by indicating the number of lactide and l-Phe units incorporated in the polymer chain with subscripts. Synthesis of PLLA_25_-*b*-PPhe_13_ is reported as an example of the methodology.

Amino-functionalized polylactide initiator (305 mg, 0.085 mmol) was weighed in a sealable tube. The system was pumped-filled and charged with nitrogen. An anhydrous mixture of CH_2_Cl_2_-DMF (60:40, 1.25 mL) was then added under inert atmosphere. A solution of l-Phe-NCA (212 mg, 1.108 mmol) in anhydrous CH_2_Cl_2_/DMF (60:40, 1.25 mL), previously pumped-filled and charged with nitrogen, was finally added to the initiator solution. The reaction was left stirring at 40 °C for 24 h. The viscous gel obtained was poured into a large quantity of chilled diethyl ether and the solution was centrifuged three times at 10 °C, 12,000 rpm (15 min each time) to recover the polymer.

Yield: 416 mg (80%). ^1^H-NMR (300 MHz, CDCl_3_ + TFA): δ = 7.85 (s, 14H, -N*H*-), 7.34–7.15 (m, 43H, Ar*H*), 7.12–6.85 (m, 22H, Ar*H*), 5.23 (q, *J* = 7.1 Hz, 24H, -C*H*CH_3_), 4.75–4.58 (m, 13H, COC*H*NH), 2.97–2.78 (m, 26H, -C*H*_2_Ph), 1.60 (d, *J* = 7.1 Hz, 72H, -CHC*H*_3_). ^13^C-NMR (300 MHz, CDCl_3_ + TFA): δ = 171.7 (C=O, peptide block), 170.9 (C=O, polylactide block), 134.3, 129.3, 129.1, 127.9 (phenyl group), 70.1 (-*C*H-CH_3_), 55.3 (-*C*H-CH_2_-), 38.6 (-CH-*C*H_2_-), 16.4 (-CH-*C*H_3_). IR (ATR) (ν, cm^−1^): 3284 (NH st), 2997 (CH_3_ st), 2944 (CH_3_ st), 1753 (C=O st), 1631 (CO st, amide I), 1522 (CO-NH st, amide II), 1454 (CH_3_ δ), 1359 (CH_3_ δ), 1181 (C-O st), 1085 (C-C vibration), 1043 (C-C vibration), 740 (Ar C-H δ), 696 (Ar C-Hδ).

### 3.5. Characterization

^1^H- and ^13^C-NMR spectra were recorded on a Bruker AMX-300 spectrometer at 300 and 75 MHz, respectively. Chemical shifts are given in ppm relative to TMS for proton and carbon spectra. Deuterated chloroform (CDCl_3_) was used as the solvent at room temperature for amino-terminated polylactide samples. For block copolymers, small amounts of TFA were required to be added in order to solubilize the sample.

Infrared absorption spectra were recorded with a Jasco FTIR 4100 Fourier Transform spectrometer. An MKII Golden Gate attenuated total reflection (ATR) accessory from Specac was used.

MALDI-TOF-MS was performed on a 4800 *Plus* MALDI TOF/TOF analyzer (ABSciex, 2010) operating in reflection mode. A MALDI solid state laser (Nd:YAG) (355 nm, 200 Hz, 3–7 ns pulse) was used as ionization source. The spectra were obtained in the positive ion mode. The samples were prepared by the dried droplet technique as follows: 2 mg of copolymer was dissolved in 50 µL of concentrated TFA. A mixture of 10 µL of dithranol (27 mg/mL THF) and 1 µL of potassium trifluoroacetate (salt additive) (5 mg/mL THF) was used as a matrix. One µL of the mixture of polymer solution/matrix solution 1/1 (*v*/*v*) was spotted on the MALDI sample plate and allowed to dry at ambient temperature before analysis.

Molecular weight was estimated by gel permeation chromatography (GPC) using a liquid chromatograph (Shimadzu, model LC-20AD) equipped with LC solution GPC software (Shimadzu). A PL HFIP gel 9 µm column (Polymer Lab 300 × 7.5 mm) at 40 °C and a refractive index detector (Shimadzu RID-10A) were employed. The polymer was dissolved and eluted in 1,1,1,3,3,3-hexafluoroisopropanol containing CF_3_COONa (0.05 M) at a flow rate of 1 mL/min (injected volume 20 μL, sample concentration 2–6 mg/mL). The number and weight average molecular weights and molar-mass dispersities were calculated using poly(methyl methacrylate) standards.

X-Ray powder diffraction patterns were obtained with a PANalytical X’Pert PRO MPD *θ*/*θ* powder diffractometer with Cu K_α_ radiation (λ = 1.5418 Å) and a silicium monocrystal sample holder. Operating voltage and current were 40 KV and 50 mA, respectively. Thin samples sandwiched between low absorbing films were used.

UV-vis spectra were recorded with a UV-visible spectrometer (Shimadzu 3600) in the 200–400 nm range from polymer solutions diluted in 1,1,1,3,3,3-hexafluoroisopropanol.

Differential scanning calorimetry analyses (DSC) were carried out using a TA Instruments Q100 series at a rate of 20 °C/min under nitrogen. DSC calibration was performed with indium. Thermal degradation was studied at a heating rate of 10 °C/min with around 5 mg samples using a Q50 thermogravimetric analyzer of TA Instruments and under a flow of dry nitrogen. Test temperatures ranged from 30–600 °C.

## 4. Conclusions

Amino-functionalized polylactide macroinitiators were effective to obtain hybrid copolymers constituted by polyphenylalanine blocks. Solubility characteristics allowed verifying the covalent linkage between highly soluble polyester blocks and poorly soluble peptide blocks. Analysis of NMR spectra was useful to estimate the length and molecular weight of the peptide blocks incorporated into the hybrid copolymer. Peptide blocks increased the thermal stability of amino-terminated PLLA samples, whereas their decomposition was enhanced with increasing the lactide content in the hybrid copolymer.

GPC chromatograms of the most soluble fractions clearly indicate that copolymers were able to form high molecular weight aggregates in 1,1,1,6,6,6-hexafluoroisopropanol solutions.

X-ray diffraction data indicate that polyester blocks were easily crystallized according to the α-form of PLLA where segments adopted a 10_7_ helical conformation. Diffraction profiles also show a crystalline arrangement of PPhe blocks, which could not be detected in DSC heating traces because thermal degradation took place before their fusion. Peptide aggregates were effective nucleating agents that influenced the cold crystallization process of PLLA blocks and the spherulitic morphologies obtained from melt crystallization. Experimental data indicate that polyester and peptide blocks tended to organize independently, thus preserving their inner physical properties. UV measurements performed in HFIP solution also showed that the peptide fragment retained the conformational preferences of l-phenylalanine since no differences were detected in the corresponding absorption peak wavelengths. The studied systems may be interesting for further investigations involving peptide-guided assemblies.
